# Acute Leukemia of Ambiguous Lineage: Diagnosis and Evaluation by Flow Cytometry

**DOI:** 10.3390/cancers17050871

**Published:** 2025-03-03

**Authors:** Franklin Fuda, Weina Chen

**Affiliations:** Department of Pathology, The University of Texas Southwestern Medical Center, Dallas, TX 75390, USA

**Keywords:** acute leukemia of ambiguous lineage, mixed-phenotype acute leukemia (MPAL), AUL, lineage, immunophenotype, genetics, diagnosis

## Abstract

Acute leukemia of ambiguous lineage (ALAL) includes mixed-phenotype acute leukemia (MPAL), which exhibits immunophenotypic evidence of differentiation along more than one cell lineage, and acute undifferentiated leukemia (AUL), which lacks sufficient immunophenotypic differentiation along any cell lineage. This review provides an overview of ALAL, emphasizing the central role of flow cytometric analysis in its diagnostic workflow. It primarily focuses on MPAL, addressing updated classification and diagnostic criteria by the WHO-HEM5 and the ICC, including both genetically defined and phenotypically defined MPAL. The article provides a detailed review of the MPAL lineage assignment criteria with an illustrative description of a series of MPAL cases. Future studies are needed to reconcile the different criteria used in these two classifications. Continuously expanded molecular studies are expected to provide a genomic and lineage-associated framework for the classification of ALAL with clinical relevance in the diagnosis and therapy selection.

## Simple Summary

This review provides an overview of acute leukemia of ambiguous lineage (ALAL), emphasizing the central role of flow cytometric analysis in its diagnostic workflow. It primarily focuses on mixed-phenotype acute leukemia (MPAL), addressing updated classification and diagnostic criteria by the WHO-HEM5 and the ICC, including both genetically defined and phenotypically defined ALAL/MPAL. The article provides a detailed review of the MPAL lineage assignment criteria with an illustrative description of a series of MPAL cases and addresses future directions for refining diagnostic criteria. Continuously expanded molecular studies are expected to provide a genomic and lineage-associated framework for the classification of ALAL with clinical relevance in the diagnosis and therapy selection.

## 1. Introduction

Acute leukemia of ambiguous lineage (ALAL) represents a subset of acute leukemia that does not show definitive differentiation along a single hematopoietic lineage. This category includes mixed-phenotype acute leukemia (MPAL), which exhibits immunophenotypic evidence of differentiation along more than one cell lineage (such as B-lineage, T-lineage, and/or myeloid lineage), and acute undifferentiated leukemia (AUL), which lacks sufficient immunophenotypic differentiation along any cell lineage [1,2,3,4]. Overall, ALAL accounts for less than 4% of total acute leukemia. It is recognized in the 5th edition of the World Health Organization Classification of Tumors of the Hematopoietic and Lymphoid Tissues (WHO-HEM5) and the International Consensus Classification (ICC).

The WHO-HEM5 and ICC classifications for ALAL are very similar, with a few differences, as noted in the discussion below. Both classifications emphasize integrating immunophenotypic, cytogenetic, and molecular findings, as well as clinical features, to diagnose ALAL and distinguish it from other acute leukemias. Among methodologies, flow cytometric immunophenotyping plays a crucial role as the cornerstone of immunophenotypic analysis. This review provides an overview of ALAL, with emphasis placed on the central role of flow cytometry in its diagnostic workflow. It will primarily focus on MPAL, addressing updated classification and diagnostic criteria, including both genetically defined and phenotypically defined MPAL. Additionally, it will address future directions for refining diagnostic criteria.

## 2. Update on ALAL/MPAL Diagnosis

Diagnosis of MPAL and AUL requires a comprehensive approach that integrates morphological and immunophenotypic characteristics (lineage attributes), cytogenetic and molecular features (biological attributes), and clinical history (clinical attributes).

All ALAL categories must meet the following criteria:

(1) The total number of neoplastic blasts in the blood or bone marrow must be ≥20% of the total cells based on morphological evaluation.
(2) Blasts in MPAL show differentiation along more than one lineage that meets MPAL lineage-defining criteria (as outlined in Table 1). In contrast, blasts in AUL lack sufficient immunophenotypic differentiation along any cell lineage.
(3) The leukemia cannot be classified into other clinically or genetically defined categories.

According to the WHO-HEM5/ICC [1,2,3], leukemias of mixed phenotype that can be assigned to other clinically or genetically defined categories should not be diagnosed as MPAL despite meeting the lineage criteria, as described below (immunophenotypic section). These include acute myeloid leukemia (AML) with defining genetic abnormalities (such as *RUNX1*::*RUNX1T1* and *CBFB*::*MYH11* fusion), chronic myeloid leukemia (CML) in blast crisis, myeloid/lymphoid neoplasms with eosinophilia and kinase gene fusions (such as *FGFR1*-rearranged myeloid/lymphoid neoplasms), therapy-related AML, and AML with myelodysplasia-related changes (AML-MR). Such cases should be classified by their specific classifications rather than labeled as MPAL, with a note that they exhibit a mixed-lineage immunophenotype. Among these clinically or genetically defined categories, the exclusion of leukemia of mixed phenotype with myelodysplasia-related (MR) changes from the MPAL classification remains highly controversial. Notably, the criteria for MR changes have been modified in both the WHO-HEM5 and ICC. Both classifications add MR gene mutations and modify/expand MR cytogenetic abnormalities. The impact of these changes on the boundary between AML-MR and MPAL and on MPAL diagnosis requires clarification in future studies [5,6].

Diagnosis of AUL takes a similar general approach as MPAL; however, in AUL, the leukemias show no clear evidence of differentiation along any established lineage based on comprehensive immunophenotypic analysis using the markers covering common and rare/unusual neoplasms (such as plasmacytoid dendritic, erythrocytic, megakaryocytic, and basophilic cell lineages).

## 3. Update on ALAL/MPAL Classification

ALAL is subclassified into two main groups by both the WHO-HEM5 and ICC based on (1) defining genetic abnormalities and (2) defining immunophenotypic changes (Table 1) [1,2]. The WHO-HEM5 includes an additional rare entity (i.e., MPAL, T/megakaryocytic) within the “MPAL defined immunophenotypically” subgroup that the ICC does not recognize. As seen in Table 1, the WHO-HEM5 uses the term ALAL rather than MPAL to label its broad categories, as well as leukemia associated with *BCL11B* rearrangement. Otherwise, the classification categories for MPAL essentially mirror each other.

## 4. Update on Immunophenotypic Criteria in Diagnosing MPAL

Flow cytometric (FC) immunophenotyping plays an essential role in diagnosing MPAL by determining lineage and immunophenotypic aberrancies. Lineage assignment involves evaluating the intensity and pattern of expression of lineage-defining antigens, such as myeloperoxidase (MPO), CD3, and CD19 (along with other B-lineage markers) on the malignant blast population relative to normal counterparts. Identifying immunophenotypic aberrancies and deviations relative to normal counterparts is necessary to avoid mistaking expansions of normal progenitors as a part of the leukemia population in the MPAL. Normal progenitors should not be included in the overall blast count. Cytochemical stains for MPO and nonspecific esterase (NSE) may be used to demonstrate myeloid lineage in conjunction with flow cytometric immunophenotyping. Immunohistochemistry may also be used to demonstrate expression of lineage-associated antigens, although discriminating normal from leukemic populations may be difficult in a case with only a small population of neoplastic blasts.

In this review article, we define two types of markers in diagnosing ALAL/MPAL, *lineage-defining* and *lineage-associated* markers. *Lineage-defining* markers (Table 2) are the markers used to assign the lineage commitment and differentiation (such as CD19, CD3, and MPO), whereas *lineage-associated* markers are the markers commonly expressed but not specific for the lineage commitment (such as CD7, CD13, and CD33).

### 4.1. Biphenotypic Versus Bilineal MPAL

**Figure 1:**
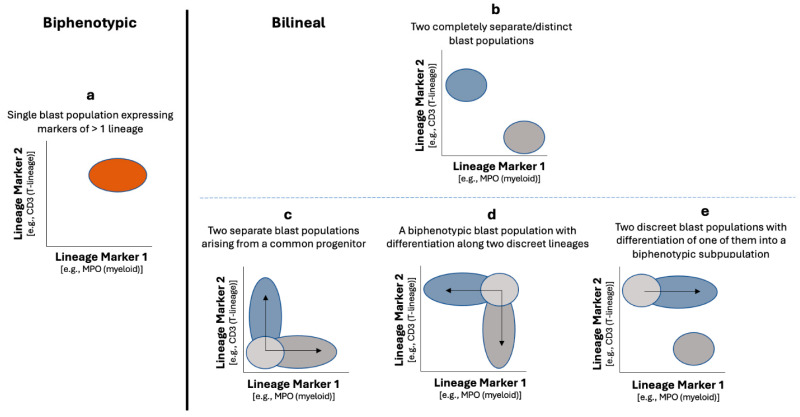
MPAL, biphenotypic vs. bilineal leukemia. Diagram plots recreated and adapted from Dr. Brent Wood’s Presentation on “Flow Cytometric Analysis of MPAL: Update on the WHO Classification”, Society for Hematopathology Grand Rounds, 3 May 2023 [6]. Biphenotypic leukemia (**a**) contains a single blast population that shows evidence of differentiation along more than 1 lineage. Bilineal leukemia contains >1 blast population that can show various combinations of differentiation along >1 lineage. Bilineal leukemia may contain multiple distinct populations of blasts (as seen in (**b**)) and/or less distinct subpopulations of blasts (common examples including categories (**c**–**e**)). However, not all cases of MPAL will fit perfectly into one of these patterns, and other patterns may be observed. The most common pattern of bilineal leukemia contains two subpopulations of blasts, likely arising from a common progenitor (**c**).

**Figure 2:**
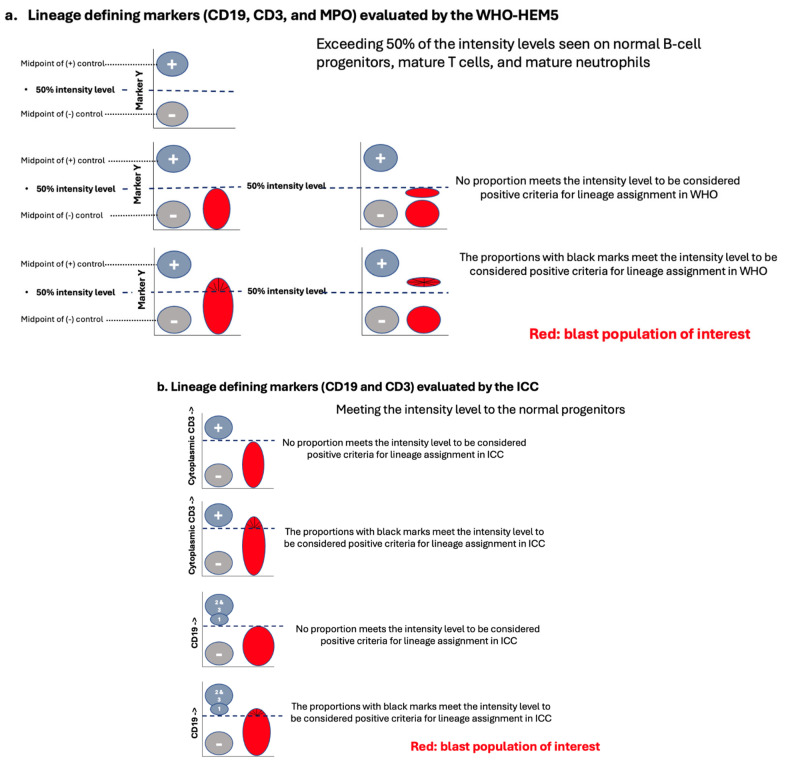
Expression intensity thresholds to determine significant expression of markers for lineage assignment. (**a**) Criteria set by the WHO-HEM5. The marker being evaluated is on the Y-axis. An expression intensity that reaches 50% of the expression intensity of a designated internal positive control population by any fraction of the aberrant blast population is considered significant expression for lineage commitment. To establish the 50% intensity threshold point, we first establish the dynamic range of expression for a given antigen on a log or biexponential scale. To do this, the midpoint of the expression intensity for a defined positive internal control population and the midpoint of the spread of a defined negative internal control population are determined. The dynamic threshold lies between these two lines. The 50% intensity threshold is then set at the midpoint of this dynamic threshold. The defined internal (+) control populations include normal B-cell progenitors for B-lineage, mature T-lymphocytes for T-lineage, and neutrophils for myeloid/non-monocytic lineage. (**b**) Criteria set by the ICC for B-lineage and T-lineage assignment. For B-lineage, a fraction of the aberrant blast population must reach the level of expression intensity seen on stage 1 B-cell progenitors (i.e., stage 1 hematogones) for the various B-lineage markers evaluated. The ovals are labeled for the different stages of B-cell progenitors. For T-lineage, a fraction of the aberrant blast population must reach the level of expression intensity of cytoplasmic CD3 expression by mature T-lymphocytes.

**Figure 3:**
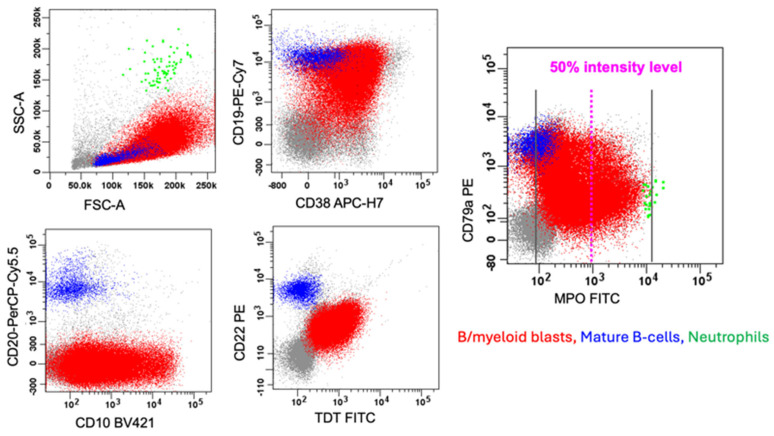
MPAL with *P2RY8::CRLF2* fusion, B/myeloid (biphenotypic, pattern 1a). This figure illustrates an example of a biphenotypic leukemia (reported recently in [8]) with a single blast population showing differentiation along more than one lineage (biphenotypic, pattern 1a). Blue represents normal, mature B-lymphocytes. Red represents the biphenotypic blast population. This biphenotypic population meets both the WHO-HEM5 and ICC criteria for B-lineage assignment with strong expression of CD19, along with strong expression of CD10, CD22, and cCD79a, and for myeloid lineage assignment with strong expression of MPO (intensity in a part of blasts exceeding 50% of the mature neutrophil level). The enlarged plot shows the midpoint (i.e., 50% intensity level in a log-axis) of the dynamic range for MPO expression established using the midpoint of the neutrophils (green) as the positive control and the mature B-lymphocytes as the negative control.

**Figure 4:**
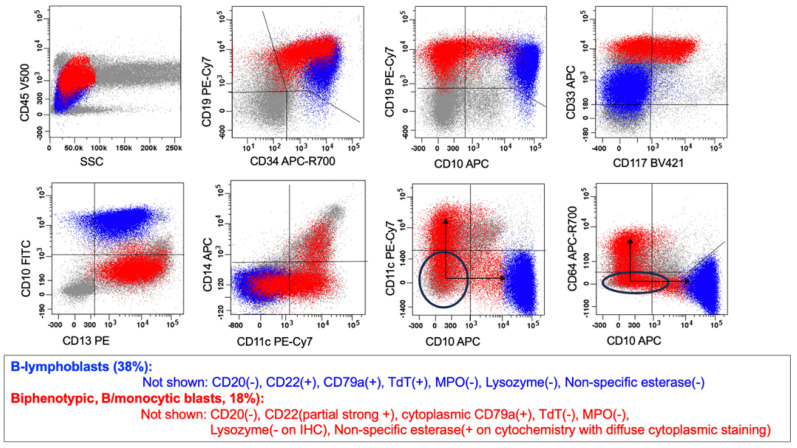
MPAL with *BCR::ABL1* fusion, B/myeloid-monocytic (bilineal, pattern 1c). This figure illustrates an example of a bilineal leukemia with two malignant hematopoietic blast subpopulations with a common progenitor (Figure 1, pattern 1c). The blue population represents malignant B-lymphoblasts. The red population represents malignant biphenotypic blasts with evidence of both B-lineage and monocytic lineage differentiation and what appears to be a common progenitor (see the ovals). The malignant B-lymphoblast population shows arrested maturation at a CD34(+) stage and aberrant expression of the myeloid-associated markers CD13 and CD33. The malignant biphenotypic blast population meets criteria for B-lineage assignment (i.e., strong CD19 along with strong CD22 and cCD79a, the latter two are not shown) and for monocytic lineage assignment (by a coordinated expression of multiple monocytic markers, CD11c, CD14, and CD64, on a subset of blasts). Nonspecific esterase (NSE) was also expressed (not shown).

MPAL can consist of either a single population of biphenotypic blasts (biphenotypic) or a mixture of various immunophenotypically aberrant blast populations (bilineal or trilineal). In biphenotypic MPAL, there is a single blast population that expresses markers of more than one lineage and meets the lineage-defining criteria for MPAL blasts (Table 2, diagrams in Figure 1a and Figure 2, and an example case in Figure 3). Conversely, in bilineal or trilineal MPAL, there is more than one type of neoplastic blast population, such as neoplastic B-lymphoblasts combined with neoplastic myeloblasts, and these subpopulations of blasts may demonstrate variable expression patterns (diagrams in Figure 1b–e, and an example case in Figure 4). The most common expression pattern consists of a subpopulation of cells with immunophenotypic features, indicating a shared progenitor, leading to the emergence of two distinct subpopulations (diagrams in Figure 1c and Figure 4) [7].

### 4.2. When and How to Apply the Lineage-Defining Criteria in MPAL Diagnosis

Determining whether MPAL is subclassified as biphenotypic, bilineal, or combined bilineal/biphenotypic can sometimes be straightforward, but it may also present challenges. While neither classification scheme requires this distinction to be specified in the final diagnosis, identifying the different subsets of neoplastic blasts can be crucial when deciding whether to apply MPAL lineage assignment criteria (Table 2) to establish a diagnosis of MPAL.

#### 4.2.1. MPAL, Bilineal/Trilineal with Two or More Separate Discrete Blast Populations (Figure 1, Pattern 1b)

Among the various patterns of MPAL illustrated in Figure 1a–e, only pattern 1b is defined by separate, distinct populations of single lineage blasts. The blasts in this particular pattern are not required to meet the MPAL lineage assignment criteria for blasts in MPAL diagnosis. Instead, any neoplastic single lineage blast population only needs to meet the criteria for single lineage acute leukemia. For instance, there is no requirement for MPO expression in myeloblasts of acute myeloid leukemia (AML). Similarly, neoplastic myeloblasts in pattern 1b are not required to demonstrate MPO expression in order to be classified as myeloid lineage. As long as the combined malignant blasts from different lineages constitute at least 20% of the total cells based on morphology, these cases can be appropriately classified as MPAL by immunophenotype. However, there is a difference between the WHO-HEM5 and the ICC regarding whether there is a minimum percentage for each neoplastic blast component. The WHO-HEM5 permits the designation of MPAL regardless of the population size of the individual aberrant blast population (i.e., no minimum percentage for either blast component) as long as blasts of either component demonstrate immunophenotypic aberrancy to discriminate them from corresponding normal precursors [1,9,10]. In contrast, the ICC stipulates that the smaller aberrant blast population must constitute at least 5% of the total cells. If this smaller population falls below 5%, the ICC recommends classifying the leukemia based on the predominant leukemic blast population and adding a descriptive modifier for the minor population. For example, if there is a composition of 30% neoplastic B-lymphoblasts and 4% neoplastic myeloblasts, the leukemia would be characterized as “B-lymphoblastic leukemia/lymphoma (B-ALL) with a small leukemic population of myeloid lineage detected of uncertain significance”, per the ICC criteria [2]. In contrast, it would be designated “MPAL, B/myeloid” under the WHO-HEM5 guidelines [1].

**Figure 5:**
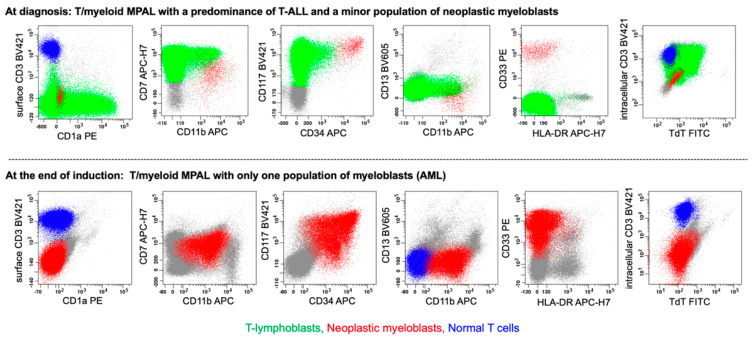
MPAL, T/myeloid (bilineal) MPAL with T-lineage predominance and a minor population of neoplastic myeloblasts at diagnosis (row 1, bilineal, pattern 1b), with persistence of the neoplastic myeloblasts (AML) at the end of induction. At diagnosis, there was a large population (78% of the total cells) of T-lymphoblasts and a minor population (0.8% of the total cells) of neoplastic myeloblasts. T-lymphoblasts (in green) are positive for CD1a (partial), CD7, CD117, CD11b (small subset), cytoplasmic CD3 (cCD3), and TdT, but negative for surface CD3 (sCD3), CD34, CD13, CD33, or HLA-DR. Neoplastic CD34+/CD117+/CD33+ myeloblasts (in red) have an aberrant immunophenotype with aberrant expression of CD7/CD11b and loss of CD13/HLA-DR. At the end of induction, AML is predominant, and T-ALL is undetectable. Notably, the neoplastic myeloblasts at the end of induction retained the same immunophenotype present at T/myeloid MPAL diagnosis, displaying aberrant expression of CD7/CD11b along with loss of CD13/HLA-DR. Color code: green, T-lymphoblasts; red, neoplastic myeloblasts; blue, normal T cells.

Whether using the ICC or the WHO-HEM5 guidelines, it is essential to document a minor population of leukemic blasts from a second lineage, as this population may become predominant after therapy. The following examples illustrate this scenario. The first example involves a case of T/myeloid MPAL, characterized by a predominance of T-cell lymphoblastic leukemia (T-ALL) along with a small population of neoplastic myeloblasts (accounting for 0.8% of the total cells) at diagnosis; at the end of the induction chemotherapy, the leukemia progressed into AML (Figure 5). In a second case recently reported in the literature, a B/T MPAL displayed a predominance of B-lymphoblasts, with a minor population of malignant T-lymphoblasts (accounting or 1.8% the total cells) at diagnosis—this case ultimately relapsed as T-ALL [11]. These two examples highlight how a minor leukemic population can have significant therapeutic and prognostic implications. It is also essential to document the dominant blast population in MPAL, as this information may affect the clinical decision regarding the choice of induction chemotherapy—whether to use ALL-based or AML-based regimens. At our institution, we follow the WHO-HEM5 criteria in these situations and specify the predominant population, e.g., T/myeloid MPAL with a predominance of T-lineage differentiation (Figure 5).

#### 4.2.2. MPAL, Biphenotypic/Bilineal of Other Patterns

For MPAL with other patterns (1a and 1c to 1e, either bilineal or biphenotypic), it is essential to apply MPAL lineage assignment criteria for the neoplastic blasts when using either the WHO-HEM5 or the ICC. When implementing these criteria, evaluating the intensity of expression for lineage-defining markers is a vital part of the assessment. A moderate to high intensity of these markers is more indicative of true lineage differentiation compared to low-intensity expression. According to the WHO-HEM5, meaningful expression for lineage assignment requires exceeding 50% of the intensity levels of the normal counterparts (Figure 2a and an example case in Figure 3). Similarly, the ICC stipulates that moderate to strong expression intensity is necessary for assignments related to B-lineage and T-lineage (refer to the discussion below and Figure 2b). Neither the WHO-HEM5 nor the ICC classification provide specific guidelines on the cut-off for the percentage of positive cells.

The criteria for assigning lineage in MPAL by the WHO-HEM5 and ICC share many similarities, although there are notable differences that include the slightly different definition of strong lineage-defining antigen expression (Table 2 and Figure 2). Moreover, the WHO-HEM5 also emphasizes the heterogenous pattern of antigen expression to enhance the specificity of lineage assignment, by demonstrating antigen expression recapitulating maturational expression similar to their non-leukemic cellular counterparts/normal progenitors and a coordinated pattern of expression of multiple antigens along the same lineage. For example, the combined and coordinated expression of myeloid markers (such as CD13 and CD117) on MPO-expressed blasts is more strongly associated with myeloid lineage commitment than the expression of each antigen individually. The ICC does not specify any pattern of antigen expression.

### 4.3. B-Lineage Assignment

Both classifications require a combination of specific B-lineage-defining markers for B-lineage assignment, with commonly assessed markers including CD19, CD22, CD79a, and CD10. The evaluation process typically starts with CD19. Strong expression of CD19 necessitates the presence of at least one of the other three markers for B-lineage assignment. In cases where the blast population shows weak CD19 expression, positive expression of at least two of the other markers becomes essential. The determination of “strong” versus “weak” expression is based on the intensity of antigen expression rather than the percentage of positive cells. According to the WHO-HEM5, expression is classified as strong if any part of the neoplastic blast population exceeds 50% of the intensity level observed in normal B-cell progenitors (as illustrated in Figure 2a and Figure 3). Conversely, the ICC defines strong expression as occurring when any part of the neoplastic blast population reaches the intensity level of CD19 seen in stage 1 B-cell progenitors (also shown in Figure 2b) or mature B cells [2]. Cases that do not contain normal B-cell progenitors are commonly encountered. The ICC addresses this issue by considering mature B cells as an acceptable positive reference population for cases that lack B-cell precursors. The WHO-HEM5 guidelines do not provide a solution for this situation. However, the median expression of CD19 in the three stages of B-cell precursors is very similar to that of mature B-lymphocytes. Therefore, using the median expression of mature B-lymphocytes seems reasonable when following the WHO-HEM5 criteria.

There are slight discrepancies between the WHO-HEM5 and ICC regarding the additional three B-lineage-associated markers. The WHO-HEM5 requires that these additional markers exhibit strong expression, regardless of whether CD19 expression is strong or weak. In contrast, the ICC mandates strong expression of the additional markers only when CD19 expression is weak. Importantly, the WHO-HEM5 excludes the use of CD79a in B-lineage assignment if the aberrant blast population meets the criteria for T-lineage assignment due to the frequent aberrant expression of CD79a in T-lymphoblastic leukemia/lymphoma. The ICC does not specify any such exclusion regarding CD79a.

The WHO-HEM5 briefly mentions using PAX-5 through immunohistochemical evaluation for B-lineage assignment, noting that its lineage specificity in cases of ALAL or MPAL is not well established [1,3]. The ICC includes PAX5, OCT2, and BOB1 in its table of markers used for lineage assignment but does not provide specific guidelines on their application [2]. Additionally, the WHO-HEM5 indicates that B-lineage assignment may still be established without CD19 expression if PAX5, CD79a, and CD22 are present, although it does not clarify the required intensity or strength of expression for these markers.

### 4.4. T-Lineage Assignment

Unlike B-lineage assignment, T-lineage assignment requires only the expression of a single cell marker, CD3. Flow cytometric evaluation of CD3 expression is most effectively measured using bright fluorophores, such as PE, APC, or BV dyes [3]. Similar to evaluating for B-lineage assignment, there are notable differences between the WHO-HEM5 and the ICC in evaluating CD3 expression. The WHO-HEM5 permits either surface or cytoplasmic expression of CD3, using a CD3 epsilon-chain antibody to meet the criteria. It requires strong expression, defined as an intensity of expression on a proportion of the blasts beyond 50% of the intensity level of that of normal mature T-lymphocytes (Figure 2a). Within the text, the ICC specifies that cytoplasmic CD3 expression must be identified, with at least a proportion of the aberrant population exhibiting an intensity comparable to that of background mature T-lymphocytes. However, the ICC table also lists surface CD3 as part of the acceptability criteria (Figure 2b). Additionally, the WHO-HEM5 permits the investigation of CD3 through immunohistochemistry (IHC), provided that a non-ζ-chain reagent is utilized [1,3]. However, the ICC does not address CD3 evaluation through IHC.

### 4.5. Myeloid Lineage Assignment

In both the WHO-HEM5 and the ICC classifications, the assignment of myeloid/non-monocytic lineage relies on the expression of a single cell marker: myeloperoxidase (MPO). While this may seem straightforward, assessing adequate MPO expression for myeloid lineage commitment can be challenging due to the variability seen in MPO expression patterns across different leukemia cases. Consequently, the WHO-HEM5′s criteria for lineage assignment based on MPO expression remain somewhat ambiguous and subject to interpretation in certain cases (see below). The ICC does not provide specific threshold criteria for MPO positivity.

**Figure 6:**
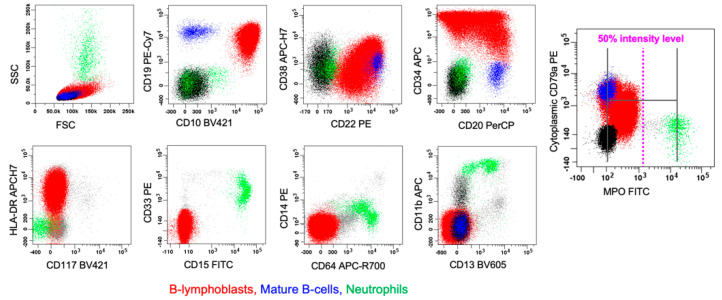
Single lineage leukemia (B-ALL) with aberrant expression of a different lineage marker (dim MPO). This figure illustrates an example of B-ALL that aberrantly expresses MPO with dim intensity (B-ALL^dim MPO^). This would not meet criteria for MPAL in the WHO classification. Red represents B-lymphoblasts, blue represents mature B-lymphocytes, black represents mature T-lymphocytes, and green represents granulocytes. The enlarged plot shows the midpoint (i.e., 50% expression intensity) of the dynamic range for MPO expression, established using the midpoint of the neutrophils as the (+) control and the mature lymphocytes as the (−) control. While a large proportion of the B-lymphoblasts show MPO expression, no proportion of the B-lymphoblasts reach the 50% intensity level. Furthermore, there is no concurrent expression of other myeloid lineage markers and no maturation pattern similar to maturing myeloid populations on the lymphoblast population. Not shown, the B-lymphoblasts lack expression of all monocytic lineage markers.

According to the WHO-HEM5, the hallmark of myeloid/non-monocytic lineage is the expression of MPO on aberrant blasts, particularly when the intensity of this expression exceeds 50% of what is observed in mature neutrophils, and/or when MPO expression is variable in a manner reminiscent of normal CD34-positive myeloid progenitors [1]. The WHO considers dim MPO expression (i.e., not reaching the 50% intensity threshold of neutrophils) to be controversial, especially in cases where the immunophenotype of the aberrant blast population aligns with that of B-lymphoblastic leukemia/lymphoma (i.e., the blast population shows relatively homogenous expression of B-lineage markers). In such instances, dim MPO expression may be uniform across the entire B-lymphoblast population or only present in a subset. This dim expression is generally not considered lineage-specific and thus does not support a myeloid lineage assignment. In both the WHO-HEM5 and the ICC classifications, these cases would typically be classified as B-lymphoblastic leukemia/lymphoma (B-ALL) with dim MPO expression. In our practice, we label these cases as “B-ALL with aberrant dim expression of MPO” (B-ALL^dim MPO^; Figure 6) [12,13].

The WHO-HEM5 entertains the idea that dim, variable MPO expression can be considered more indicative of myeloid lineage if a leukemic subset exhibits increased light-scatter properties compared to the remainder of the leukemic population. Additionally, this subset must demonstrate coordinated expression of other myeloid-associated antigens (e.g., CD117 or bright CD13/CD33), which distinguishes them from the remaining leukemic population, which exhibits more lymphoid characteristics [3]. Lineage assignment in such cases is complicated by the fact that B-ALL often shows aberrant expression of myeloid antigens, such as CD13 and CD33. To streamline the evaluation of MPO, we generally refer to Table 2a on the lineage assignment criteria for MPAL provided by the WHO-HEM5 [1,3]. This table clearly states that myeloid lineage assignment is triggered when the “intensity in part exceeds 50% of mature neutrophil level” (Table 2a and Figure 2a) [11]. If these criteria are not met, we designate the leukemia based on its single lineage determination while noting that a proportion of blasts express multiple myeloid lineage markers, including dim and variable MPO, which do not meet the current criteria for biphenotypic blasts.

Isolating mature neutrophils from those at earlier stages of maturation can be challenging, particularly when the panel lacks markers to distinguish maturity, such as CD10 or CD16. Mature neutrophils exhibit a higher intensity of MPO expression compared to earlier-stage cells. Therefore, setting the median for a positive MPO control may be skewed if we include the entire granulocyte population. Since mature neutrophils express a slightly higher intensity level of CD45 compared to earlier-stage neutrophils, we typically rely on the proportion of granulocytes demonstrating slightly higher expression of CD45, while being cautious to limit contamination from eosinophils, to serve as our internal positive control.

In addition to strong MPO expression, myeloid lineage assignment can also be fulfilled by showing evidence of monocytic differentiation. In both WHO-HEM5 and ICC classifications, this is accomplished by identifying the expression of more than one of five monocytic-associated markers (i.e., CD11c, CD14, CD64, lysozyme, and/or nonspecific esterase; Table 2). The WHO-HEM5 specifies that cytochemical staining for NSE must show diffuse cytoplasmic reactivity associated with monocytic lineage versus the dot-like staining associated with lymphoblasts [3]. Neither classification scheme offers quantitative or qualitative thresholds for positivity of the remaining markers. Although they are not part of the five monocytic markers associated with MPAL classification in the WHO-HEM5 or the ICC guidelines, we frequently observe varying levels of CD11b, CD15, and CD36 expression, along with a notably bright intensity of CD33 on the monocytic components in MPAL.

## 5. ALAL/MPAL with Defining Genetic Abnormalities

Distinct subsets of ALAL are categorized based on their defining genetic alterations. This subcategory previously included MPALs with *BCR::ABL1* fusion and *KMT2A* rearrangements. It has now been expanded to include two molecularly defined ALALs/MPALs associated with *ZNF384* rearrangements and *BCL11B* alterations [1,2]. Notably, all of these genetic abnormalities can present as distinct single lineage leukemias (e.g., B-ALL with *BCR::ABL1* fusion) but are classified as ALAL/MPAL based on meeting MPAL/ALAL immunophenotypic criteria (Table 2).

### 5.1. MPAL with BCR::ABL1 Fusion

MPAL with *BCR::ABL1* fusion accounts for 15–20% of all cases of MPAL, one of the most common genetically defined MPALs. Most patients are adults. The *BCR::ABL1* fusion is typically associated with a B/myeloid immunophenotype MPAL (an example case of *BCR::ABL1* rearranged B/myeloid-monocytic (bilineal, pattern 1c, in Figure 4))*,* but other immunophenotypic variants, such as AUL, T/myeloid, and B/T or B/T/myeloid MPAL, can occasionally occur [14,15,16]. It is important to ensure that a blast crisis of chronic myeloid leukemia with an MPAL immunophenotype, post-cytotoxic therapy leukemia with an MPAL immunophenotype, and MPAL that has acquired *BCR::ABL1* secondarily is not miscategorized as de novo MPAL with *BCR::ABL1* fusion.

### 5.2. MPAL with KMT2A Rearrangement

MPAL with *KMT2A* rearrangement accounts for ~10% of cases of MPAL and occurs more frequently in children. The fusion partners include *AFF1*(AF4), MLLT3(AF9), and *MLLT1*(ENL). While the WHO-HEM5 introduction clearly states that all categories of MPAL require ≥20% blasts, the subsection describing *KMT2A*-rearranged MPAL states that this subtype does not require ≥20% blasts for diagnosis; in our practice, we use the ≥20% blasts criteria.

**Figure 7:**
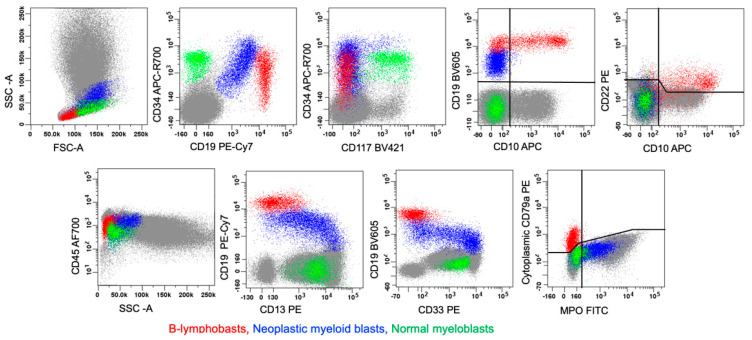
MPAL with *KMT2A::USP2* fusion, B/myeloid (bilineal, pattern 1b). This figure illustrates an example of a bilineal leukemia with two separate/discrete malignant hematopoietic blast populations. The red population represents malignant B-lymphoblasts. The blue population represents malignant myeloid blasts. The malignant B-lymphoblasts show arrested maturation at a CD34(+) stage and aberrant partial loss of CD10. These B-lymphoblasts lack expression of MPO as well as monocytic markers (i.e., CD11c, CD14, CD64, and lysozyme—not shown). The malignant myeloid blasts show aberrant expression patterns for myeloid markers CD13, CD33, CD117, and MPO (partial), and aberrant expression of the B-lineage marker CD19. This myeloid blast population lacks significant expression of CD10, CD22, and cCD79a; thus, it does not meet the WHO or ICC criteria for a biphenotypic population. The green population represents normal myeloblasts for comparison.

MPAL with *KMT2A* usually has a B/myeloid immunophenotype (an example case of MPAL with *KMT2A::USP2* fusion, B/myeloid (bilineal, pattern 1b, in Figure 7)), although other immunophenotypic variants, including rare cases of AUL and T/myeloid MPAL, have been reported [14,15,16]. Similar to B-ALL with *KMT2A* rearrangement, this MPAL often features a B-lymphoblastic component that lacks CD10 expression, and similar to AML with *KMT2A* rearrangement, the myeloid component frequently demonstrates monocytic differentiation. At our institution, we have occasionally encountered MPAL with *KMT2A* rearrangement that exhibits typical immunophenotypic features of *KMT2A*-rearranged B-ALL (i.e., decreased to negative expression of CD10 and CD24, along with positive expression of CD15), but with strong expression of MPO.

### 5.3. MPAL with ZNF384 Rearrangement

**Figure 8:**
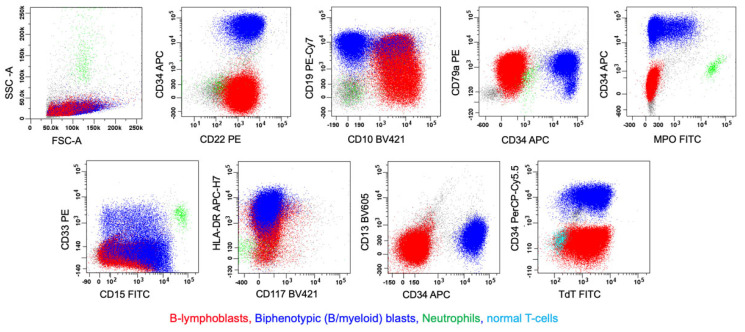
MPAL with *EP300-ZNF384* fusion (B/myeloid, bilineal/biphenotypic, pattern 1e). This is an example of a bilineal leukemia with two discreet blast populations with differentiation of one of them into a biphenotypic subpopulation. The red population represents malignant B-lymphoblasts. The blue population represents malignant B/myeloid blasts. The malignant B-lymphoblasts express CD19, CD22, CD79a, CD15, HLA-DR, and TdT, but lack CD20 (not shown), CD34 CD13, CD33, CD117, or MPO. The malignant B/myeloid blasts express CD34, CD10 (in a small subset), CD15, CD33 (partial+), CD19 (strong in a subset of blasts), CD22, CD79a, and MPO (strong in a subset of blasts), but lack CD13, CD33, or CD117. This immunophenotype meets the criteria for a B/myeloid biphenotypic population. The green and cyan populations represent normal myeloblasts and T cells, respectively, for comparison.

MPAL with *ZNF384* rearrangement accounts for 20% of MPAL cases, occurring in ~50% of pediatric B/myeloid MPAL, although this aberration has been identified occasionally in adult patients [17,18,19]. The fusion partners include *TCF3*, *EP300*, *TAF15*, and *CREBBP*, resulting in perturbation in chromatin binding and transcriptional dysregulation. *ZNF384*-rearranged B/myeloid MPAL and B-ALL have similar transcriptional profiles, suggesting a biological continuum [17,18,19,20]. Clinically, this entity may present as either B/myeloid MPAL or B-ALL, and leukemia lineage often shifts over the course of the disease [21,22]. Similar to B-ALL with *ZNF384* rearrangement, this B/myeloid MPAL often features a B-lymphoblastic component that has minimal or negative CD10 expression [20]. Figure 8 illustrates an example case of *EP300::ZNF384*-rearranged MPAL (B/myeloid, bilineal/biphenotypic with two discreet blast populations with differentiation of one of them into a biphenotypic subpopulation, pattern 1e), in which CD10 expression is diminished in B/myeloid blasts but is maintained in B-lymphoblasts.

### 5.4. ALAL/MPAL with BCL11B Rearrangement/Activation

**Figure 9:**
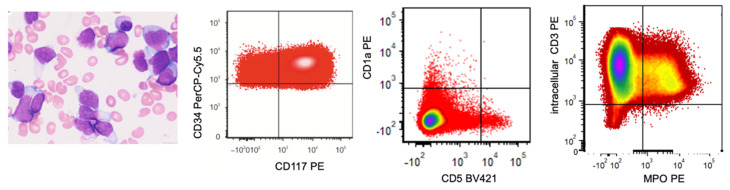
T/myeloid, MPAL with *BCL11B* activation (t(8;14)(q24.21;q32.2)/*CCDC26*::*SET3* putative fusion gene, leading to *BCL11B* deregulation due to enhancer hijacking; biphenotypic, pattern 1a). This is an example case of a biphenotypic leukemia (reported recently in [27]) with a single blast population showing differentiation along T and myeloid lineages. The left image demonstrates medium-sized to large blasts with fine chromatin and a scant to moderate amount of cytoplasm. Blasts (painted in red) express CD34, CD117, intracellular CD3, and MPO, and largely lack expression of CD1a and CD5 (Courtesy of Dr. Sanam Loghavi from The University of Texas MD Anderson Cancer Center, TX, USA).

ALAL/MPAL with *BCL11B* rearrangement/activation (r/a) accounts for 10–15% of MPAL cases, and approximately one-third of T/myeloid MPAL cases [23,24,25]. The other subtype has a more heterogenous immunophenotype, identified in AUL, AML with minimal differentiation or without maturation, and ~20–30% of ETP-ALL, which indicates biological continuums among these leukemias [23,24,25]. Rearrangements of *BCL11B* (at chromosome 14q32) with various partners (*ARID1B*, *CCDC26*, *CDK6*, etc.) reposition regulatory sequences downstream or upstream of the *BCL11B* or *BCL11B* enhancer tandem amplification (BETA). All of these alterations result in aberrantly high expression of the BCL11B protein [23,24,25,26]. Deregulation of *BCL11B* often co-occurs with alterations in *FLT3-*internal tandem duplication. Blasts in T/myeloid MPAL with *BCL11B-*r/a express hematopoietic stem cell antigens (such as CD34, CD117), express CD3 (typically cytoplasmic), MPO, and other T-lineage-associated markers (such as CD2 and CD7), and lack CD1a, CD5, and CD8 [23,25,27]. Figure 9 demonstrates a case of T/myeloid MPAL with t(8;14) (q24.21;q32.2)/putative *CCDC26*::*SET3* fusion, leading to *BCL11B* deregulation due to enhancer hijacking [27].

## 6. ALAL/MPAL Defined Immunophenotypically

Distinct subsets of ALAL that lack genetically defined abnormalities are categorized based on their immunophenotype and lineages. This subcategory includes B/myeloid MPAL, T/myeloid MPAL, rare types of MPAL (including B/T/myeloid, B/T, and T/megakaryocytic), and AUL.

### 6.1. B/myeloid MPAL

B/myeloid MPAL is the common MPAL immunophenotype, representing ~50–60% of the total MPAL cases, and possesses a heterogeneous group of gene alterations in B-lineage transcriptional regulators (*PAX5* and *IKZF1* (Ikaros)), as well as somatic mutations in epigenetic modifiers (*TET2*, *EZH2*, and *ASXL1*) [15,18]. Rare patients of B/myeloid MPAL harbor *CRLF2* rearrangement and *NUP98::NSD1* [8,17,28,29]. Blasts express both B-lineage antigens (e.g., CD19, CD22, cCD79a, and PAX5) and myeloid lineage antigens (e.g., MPO, CD13, CD14, CD15, CD64, and CD117). Biphenotypic and/or bilineage expression patterns can be seen in B/myeloid MPAL (Figure 3, biphenotypic).

### 6.2. T/myeloid MPAL

**Figure 10:**
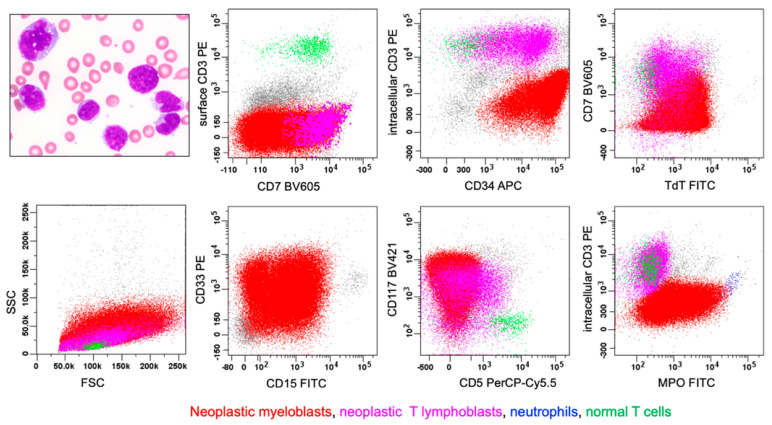
T/myeloid, MPAL (bilineal, pattern 1b, consisting of neoplastic myeloblasts and T-lymphoblasts). The upper left image demonstrates numerous myeloid-appearing blasts with fine chromatin, prominent nucleoli, and moderate amounts of cytoplasm with rare eosinophilic granules. Via flow cytometric immunophenotyping (on a BM aspirate sample), there were two neoplastic populations of blasts. Population 1 (in red) represents a 75% population of medium-sized to large myeloblasts that express CD7 (partial), CD34, TdT, CD15, CD33, CD117, and MPO, but lack expression of s/cCD3 (surface/intracellular, s/c) and CD5. Population 2 (in violet) represents a 5.7% population of medium-sized T-lymphoblasts that express CD7, cCD3, CD34, TdT, and CD117, but lack expression of sCD3, CD1a (not shown), CD5, CD15, CD33, or MPO. Mature T-lymphocytes are shown in green, and neutrophils are shown in blue.

T/myeloid MPAL is the second most common MPAL immunophenotype, representing ~20–30% of MPAL cases, and possess a heterogeneous group of gene alterations in transcription factors (*WT1*, *ETV6*, and *RUNX1*)*,* epigenetic/chromatin modifiers (*PHF6* and *DNMT3A*), *NOTCH* (*NOTCH1*), signaling pathways (JAK-STAT and RAS)*,* and fusions of *ZEB2::BCL11B* and *NUP214::ABL1* [17,19,28]. The overall genetic profiles overlap with those in T-ALL, particularly early T-precursor (ETP)-ALL [30,31]. Blasts express both T- and myeloid-lineage markers. T-lineage markers include CD3 (mostly cytoplasmic) and other T-lineage-associated markers (CD7, CD2, CD4, CD5, and CD8 in a subset, and CD1a only in a small subset). Myeloid lineage markers include MPO and other myeloid-lineage-associated antigens, such as CD13, CD15, CD33, and CD117 (an example case of T/myeloid with myeloid predominance, bilineal; Figure 10). Some cases express monocytic markers, such as CD11c, CD14, CD36, and CD64 [16,28,32,33].

### 6.3. Rare Type: B/T MPAL

B/T MPAL is rare and accounts for ∼3% of MPAL [5,11,18,19,26,28,34]. This subtype of leukemia commonly occurs in adolescents and young adults, with a male predominance and frequent involvement of PB/BM and extramedullary organs/lymph nodes [5,34]. Recurrent molecular abnormalities are overlapped with those in T-ALL, particular for those T/B MPAL with T-lineage predominance. The recurrent alterations include mutations in *NOTCH1, PHF6,* the genes involving the transcriptional regulation (*RUNX1* and *ETV6*), and the RAS (*NRAS*, *PTPN11*, and *NF1)* and JAK-STAT (*JAK3* and *IL7R*) pathways.

**Figure 11:**
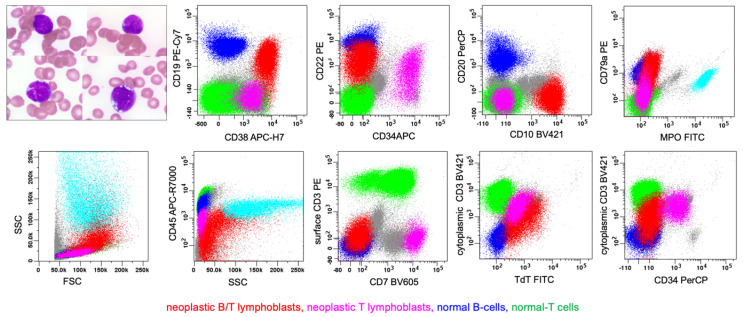
B/T MPAL (bilineal, best fitting pattern 1e, consisting of B/T-lymphoblasts and T-lymphoblasts). The upper left image demonstrates the morphologic feature of blasts in BM aspirate. There are scattered, variably sized lymphoblasts with irregular nuclei, and scant cytoplasm. A subset of blasts that are medium–large sized, with many cytoplasmic vacuoles, likely corresponding to B/T-lymphoblasts. Via flow cytometric immunophenotyping (on a peripheral blood sample), there were two neoplastic populations of blasts. Population 1 (in red) represents a 13% population of B/T-lymphoblasts that are medium to large sized with increased side scatter (SSC), that express cytoplasmic CD3 (cCD3), CD10, CD19 (dimmer than normal B cells), CD22, CD38 (not shown), CD45(partial +), CD79a, and TdT, but that lack expression of surface CD3 (sCD3), CD20 (not shown), CD34, and MPO. Population 2 (in violet) represents a 5% population of T-lymphoblasts that are medium sized with low SSC, that express cCD3, CD7, CD22 (partial), CD34, CD38, CD45(partial +), and TdT, but that lack expression of CD1a (not shown), sCD3, CD19, CD20 (not shown), CD79a, and MPO. Via immunocytochemistry (on the BM core, not shown), lymphoblasts co-expressed CD3, PAX5, and TdT. Collectively, these results support a diagnosis of MPAL, B/T, with co-predominance of B- and T-lineage cells.

Immunophenotypically, blasts co-express both B- and T-cell lineage-defining markers (an example case of B/T MPAL, bilineal, consisting of B/T-lymphoblasts and T-lymphoblasts, pattern 1e; Figure 11). Blasts express strong CD19 with strong expression of ≥1 marker, including CD22 and CD20, CD3 (cytoplasmic, a small subset of cases expressing surface CD3), CD7, TdT, and co-expressed 1–3 myeloid markers (but MPO-negative), and frequently lack CD1a [5,34]. B/T MPAL with T-lineage predominance has immunophenotypes similar to ETP-ALL (on the subset blasts with T-lineage differentiation). Of note, CD79a is not considered a B-lineage-defining marker in diagnosing B/T MPAL since CD79 can also be expressed in AML [1,2].

### 6.4. Rare Type: B/T/myeloid MPAL

B/T/myeloid MPAL is extremely rare, with only up to ~20 cases being reported in the literature. This entity compromised 7% (2/29) of the total MPAL cases in a study by Xiao et al., with gene alterations in *PHF6*, NOTCH (*NOTCH1* and *FBXW7*), and JAK/STAT pathways [28], and a molecular profile that overlapped with that in B/T MPAL and ETP-ALL. Blasts in this subtype of leukemia express B-, T-, and myeloid-lineage-defining markers, such as CD3, CD19 plus 1, or a more designated B-lineage marker, and MPO or two or more monocytic markers (Table 2).

### 6.5. Rare Type: T/megakaryocytic (T/Mk) MPAL

T/megakaryocytic (Mk) MPAL is a rare type of MPAL, with approximately 20 cases reported in the literature. It is included in the WHO-HEM5 classification but is not currently recognized in the ICC. The clinicopathological features are overlapped with those in pediatric non-Down syndrome acute megakaryoblastic leukemia (AMKL), characterized by infant onset, a male predominance, involving PB, BM, and extramedullary organs (such as the liver and spleen), harboring *CBFA2T3::GLIS2* or rarely *CBFA2T3::GLIS3*, and an extremely poor prognosis [35,36,37,38,39].

**Figure 12:**
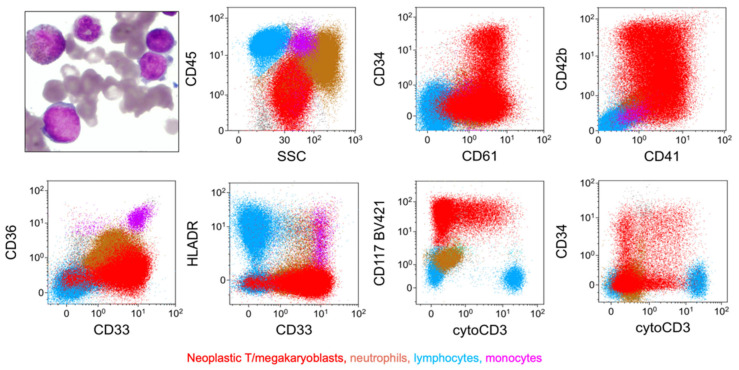
T/megakaryocytic MPAL (biphenotypic, pattern 1a) with *CBFA2T3*::*GLIS2* fusion. There is a population of variably sized blasts, some with cytoplasmic blebbing (upper-left image). Immunophenotype of blasts (red) by flow cytometry: blasts express CD34 (partial), CD41, CD42b, CD117, CD33, and cCD3 (with an intensity approaching that of normal mature T cells (blue, a part of the lymphocyte population)), and they lack CD45, CD36, or HLA-DR (Courtesy of Dr. Tembhare R. Prashant, Tata Memorial Center, Homi Bhabha National Institute (HBNI) University, Navi Mumbai, Maharashtra, India).

Blasts express both T- and megakaryocytic-lineage markers (an example case of T/Mk MPAL with *CBFA2T3*::*GLIS2* fusion, biphenotypic; Figure 12). A defining immunophenotypic feature distinguishing T/Mk MPAL from AMKL is the expression of CD3 in T/Mk MPAL; typically, cCD3 is the only T-lineage marker present with an intensity in a part of blasts approaching that of normal mature T cells. The WHO-HEM5 criteria do not specify an immunophenotypic requirement for megakaryocytic lineage assignment. It is prudent to require expression of two or more megakaryocytic markers (CD41, CD61, or CD42b) to assign this lineage. Nonetheless, future studies are needed to clarify the number of megakaryocytic markers and their intensity of expressions. Additionally, reported cases have also shown expression of myeloid markers (e.g., CD13 and CD33), and CD56, but have lacked CD36, MPO, HLA-DR, CD1a, and TdT expression [35,36,37,38,39].

### 6.6. Rare Type: B/Megakaryocytic (B/Mk) MPAL

**Figure 13:**
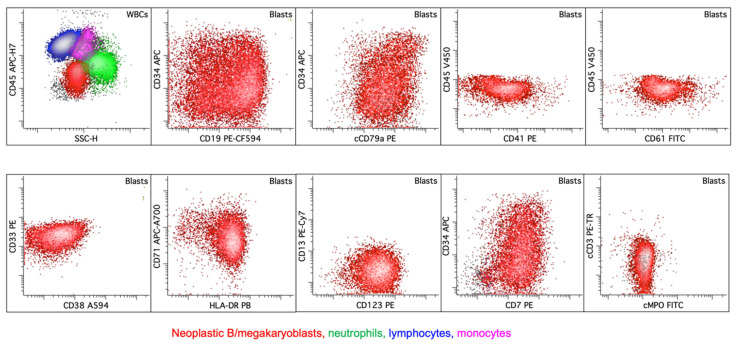
B/megakaryocytic MPAL (biphenotypic, pattern 1a). The upper-left dot plot displays total viable white blood cells (peripheral blood), with blasts shown in red, lymphocytes in blue, monocytes in pink, and granulocytic cells in green. The rest of the dot plots selectively display leukemic blasts. The leukemic blasts (colored red) have increased side scatter (SSC) and express CD7, CD19, CD34 (variable), CD79a, CD41 (variable), CD61, CD33, CD38 (dim), CD45 (decreased), CD71, CD123, and HLA-DR, without cCD3, CD13, and MPO. While not shown in the plots, the blasts also lack expression of sCD3, CD4, CD5, CD10, CD15, CD22, CD24, CD56, CD64, CD117, or CD235a (Courtesy of Dr. Xueyan Chen, University of Washington Medical Center, Seattle, WA, USA).

B/megakaryocytic (B/Mk) MPAL is a rare type of leukemia that has not yet been reported in the literature and is, therefore, not included in either the WHO-HEM5 or the ICC classifications. The clinicopathological and molecular features thus remain to be determined. Once again, it is logical that leukemic blasts should demonstrate evidence of both megakaryocytic differentiation—indicated by the expression of megakaryocytic markers, such as CD41, CD61, or CD42b (two markers expressed in an example case of B/Mk MPAL)—and B-lineage differentiation, as defined for other forms of MPAL (Table 2). Figure 13 presents a case of B/megakaryocytic MPAL (biphenotypic) with complex karyotype in a 6-year-old female with PB/BM involvement of neoplastic blasts that express CD34, TdT, B-cell markers (CD19 and CD79a), and megakaryocytic markers (CD41 and CD61), but lack cCD3/sCD3, CD13, CD15, CD117, glycophorin A, or MPO.

### 6.7. Acute Undifferentiated Leukemia

**Figure 14:**
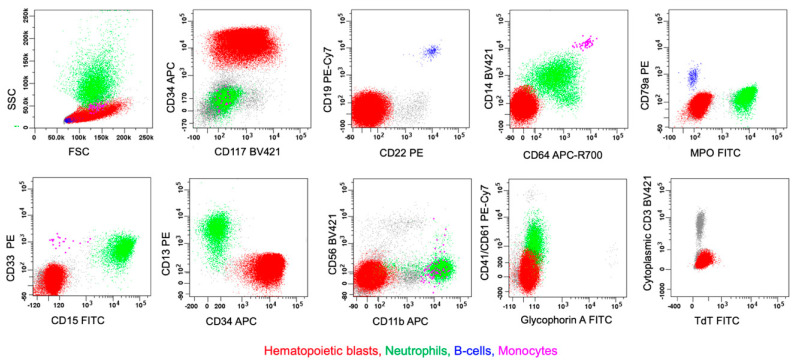
Acute undifferentiated leukemia (AUL). This figure illustrates a case of AUL in which blasts (in red) lack clear differentiation along any hematolymphoid lineage. Blasts express only CD117 (one myeloid-lineage-associated marker) and the immature cell marker (CD34, but largely lack TdT), but do not express lineage-defining markers (such as cCD3, sCD3 (not shown), or MPO), or more than one lineage-associated marker for any specific lineage (negative for CD19, CD22, CD79a, CD11b, CD13, CD15, CD33, CD56, CD41/CD61, CD64, glycophorin A, and other markers (not shown: CD11c, CD123, and HLA-DR)). This immunophenotypic profile provides no evidence of differentiation into basophilic, megakaryocytic, plasmacytoid dendritic, or natural killer (NK) lineages.

Acute undifferentiated leukemia (AUL) is a rare type of ALAL, with some cases carrying *BCL11B* rearrangement/activation [23,24]. Immunophenotypically, it is defined by the absence of clear differentiation along any hematolymphoid lineage (an example is shown in Figure 14). The blasts must not express any lineage-defining markers, such as CD3 and MPO, and they cannot express more than one lineage-associated marker for any specific lineage. Furthermore, AUL blasts must not exhibit markers that indicate basophilic, megakaryocytic, plasmacytoid dendritic, or NK lineage differentiation [1,3,40].

AUL often shows partial or complete expression of one myeloid marker, such as CD13, CD33, or CD117. Therefore, it is crucial to differentiate AUL from AML with minimal differentiation, which expresses two or more myeloid markers. Notably, in 25% to 33% of AUL cases, no myeloid markers are expressed at all. Commonly expressed markers in AUL include CD34, HLA-DR, and TdT, while the expression of CD56 is uncommon [41]. Cases that meet the immunophenotypic criteria for AUL but also exhibit cytogenetic abnormalities or molecular findings indicative of acute myeloid leukemia (AML) with myelodysplasia-related cytogenetic abnormalities or mutations should be classified as AML rather than AUL [1,2,3].

### 6.8. Acute Leukemia of Ambiguous Lineage, Not Otherwise Specified

Acute leukemia of ambiguous lineage, not otherwise specified (ALAL-NOS), is a rare subtype of ALAL. Due to the limited number of reported cases, the pathogenetic features of ALAL-NOS are not yet well established. This classification excludes cases with genetic abnormalities that are characteristic of AML or ALL, as well as cases that meet the diagnostic criteria for MPAL with defined genetic alterations. ALAL-NOS is characterized by the presence of multiple markers corresponding to a single lineage, which distinguishes it from acute undifferentiated leukemia (AUL). However, ALAL-NOS does not express specific lineage-defining markers, such as CD3, MPO, or CD19, along with additional B-lineage markers (as detailed in Table 2). Consequently, it does not meet the criteria for MPAL. Furthermore, ALAL-NOS does not fit into any single lineage classification. It may include cases that express multiple T-lineage markers but do not show surface or cytoplasmic CD3. These cases might demonstrate a few myeloid-associated antigens but lack MPO and/or markers specific to monocytic differentiation. Additionally, they may express CD19 without the presence of other B-lineage markers [1,2].

As an example, a case may completely lack B, T, and myeloid lineage defining markers while exhibiting positivity for more than one T-lineage-associated marker, such as CD2, CD5, and CD7, positivity for more than one myeloid-lineage-associated marker, such as CD13 and CD33, and positivity for only one B-lineage marker, such as CD19. The lack of T, B, and myeloid lineage defining markers (Table 2) prevents it from being classified as MPAL, T-ALL, B-ALL, and AML. Furthermore, the expression of more than one lineage-associated marker for T and myeloid lineages precludes it from being classified as AUL. Therefore, the case would be appropriately designated ALAL-NOS. 

## 7. Differential Diagnosis of MPAL from Its Immunophenotypic Mimics

There is a subtype of acute leukemias that commonly expresses cross-lineage markers or has a mixed phenotype. Awareness of this unusual immunophenotype should alert careful assessment for MPAL lineage assignment criteria and prompt cytogenetic and molecular studies to render a correct diagnosis.

### 7.1. B-ALL with Dim MPO Expression

As discussed in the above section (Section 4.2.2.), B-ALL with dim MPO has dim expression of MPO that fails to meet the 50% intensity level of mature neutrophils (Figure 6). In contrast, B/myeloid MPAL has strong expression of MPO, with at least a proportion of the blasts showing an intensity that exceeds 50% of the mature neutrophil level (Figure 3). Specificity of MPO expression on the myeloid lineage commitment could be further enhanced by demonstrating an MPO maturational expression pattern similar to their normal CD34(+) progenitor counterparts and a coordinated pattern of expression of multiple myeloid-lineage-associated antigens on the MPO-expressing cells.

### 7.2. AML with RUNX1::RUNX1T1

AML with *RUNX1::RUNX1T1* frequently expresses CD19 and PAX5. A prompt FISH (fluorescence in situ hybridization) study for *RUNX1::RUNX1T1* fusion is needed to distinguish MPAL with a B/myeloid immunophenotype from AML with *RUNX1::RUNX1T1* fusion.

### 7.3. ETP-ALL

A proportion of ETP-ALL cases, characterized by a CD3+, CD1a-, CD8-, and CD5-/weak phenotype, along with expression of myeloid-associated markers, may have immunophenotypic features that overlap with those of T/myeloid MPAL. Lack of strong MPO, and/or monocytic lineage-defining markers, distinguishes T/myeloid MPAL from ETP-ALL, which, by definition, lacks this expression. Despite this, both entities frequently harbor similar molecular alterations [30,31].

### 7.4. Myeloid and Lymphoid Neoplasm with Tyrosine Kinase Gene Fusions

Myeloid/lymphoid neoplasms with tyrosine kinase gene fusions, including *FGFR1* rearrangement, which are typically associated with eosinophilia, can present as acute leukemia with blasts showing a mixed phenotype, meeting the MPAL lineage assignment criteria. Because the kinase gene rearrangement is definitional, these leukemias should not be diagnosed as MPAL regardless of the immunophenotype.

## 8. Conclusions

The classification and understanding of acute leukemia of ambiguous lineage (ALAL) are continually evolving. There is a growing emphasis on improving the criteria for lineage-defining markers to enhance the accuracy of lineage assignment. Moving forward, it is essential to address the differences between the WHO-HEM5 classification and the ICC to establish a consensus on diagnosis. This includes clarifying the definition of strong antigen expression and determining how to manage minor populations of blasts found in bilineal MPAL. Additionally, ongoing and future molecular studies will help refine a framework that further links genomic features to lineage determination, ultimately improving the classification of ALAL and enhancing its clinical relevance for diagnosis and induction therapy selection. Finally, advancements in flow cytometry, such as new and improved fluorescent dyes, the introduction of spectral flow cytometry, and the application of machine learning tools, are paving the way for the next generation of flow cytometric immunophenotypic analysis of ALAL. While the timeline for implementing additional changes to diagnostic criteria and new technologies remains uncertain, the overall approach to flow cytometry and its relation to diagnosing ALAL will continue to evolve. This evolution aims to better meet the needs of patients and the clinicians who care for them [42,43,44,45,46,47].

## Figures and Tables

**Table 1:** Classification of acute leukemia of ambiguous lineage (ALAL) entities by the WHO-HEM5 and the ICC. Source data were acquired from [1,2].

WHO-HEM5	ICC
**ALAL with defining genetic abnormalities** MPAL with *BCR::ABL1* fusion MPAL with *KMT2A* rearrangement **ALAL with other defined genetic alterations** ALAL with *BCL11B* rearrangement MPAL with *ZNF384* rearrangement	**MPAL with defining genetic alterations** MPAL with *BCR::ABL1* MPAL with t(v;11q23.3); *KMT2A* rearranged MPAL with *BCL11B* activation MPAL with *ZNF384* rearrangement
**ALAL defined immunophenotypically** MPAL, B/myeloid MPAL, T/myeloid MPAL, B/T MPAL, B/T/myeloid MPAL, T/megakaryocytic ALAL NOS Acute undifferentiated leukemia	**MPAL with defining immunophenotypic changes** B/myeloid MPAL T/myeloid MPAL B/T/myeloid MPAL B/T MPAL ALAL NOS Acute undifferentiated leukemia

**Table 2:** Lineage assignment criteria for MPAL recreated from the WHO-HEM5 (2a) and the ICC (2b), respectively [1,2]. These are criteria to determine lineage assignment for MPAL blasts (patterns 1a and 1c to 1e in Figure 1) and are not required for MPAL with pattern 1b from Figure 1, which has discrete separate blast populations. Likewise, these criteria are not required for single-lineage leukemia (e.g., B-ALL, T-ALL, or AML).

(a) (Slightly Modified from the WHO-HEM5) *	
Lineage *	**Criterion**
**B-lineage** CD19 strong positive ^a^ or CD19 weak positive ^b^	If CD19 strong, one or more of the following is also strongly expressed: CD10, CD22, or CD79a ^c^ or If CD19 weak, two or more of the following are also strongly expressed: CD10, CD22, or CD79a ^c^
**T-lineage** CD3 (cytoplasmic or surface) ^d^	Intensity in part exceeds 50% of that of mature T cells by flow cytometry or Immunocytochemistry-positive with non-ζ-chain reagent
**Myeloid lineage** Myeloperoxidase or Monocytic differentiation	MPO intensity in part exceeds 50% of the mature neutrophil level or If two or more of these monocytic-associated markers (i.e., NSE, CD64, CD11c, CD14, or lysozyme) are expressed
^a^ CD19 intensity on at least a proportion of the population exceeds 50% of that of normal B-cell progenitors by flow cytometry. ^b^ CD19 intensity does not exceed 50% of that of normal B-cell progenitor by flow cytometry on any proportion of the blasts. ^c^ CD79a cannot be used as a criterion for B-lineage assignment if the blasts also show T-lineage differentiation. ^d^ Using CD3 epsilon-chain antibody. * Specificity on the lineage commitment is enhanced by demonstrating antigen expression recapitulating maturational expression similar to their non-leukemic cellular counterparts and a coordinated pattern of expression of multiple antigens along the same lineage.	
**(b) (Slightly Modified from ICC)**	
**Lineage**	**Markers**
**B-lineage**	
Strong CD19 ^a^ plus	≥1 marker expression of CD10, CD22, or CD79a
Weak CD19 plus	≥1 marker expression of CD10, CD22, or CD79a
Consider immunohistochemical stains for B-lineage	PAX5, OCT2, BOB1
**T-lineage**	
CD3 (surface or cytoplasmic) ^b^	
**Myeloid lineage**	
MPO or	
Monocytic differentiation	CD11c, CD14, CD64, nonspecific esterase (cytochemistry), or lysozyme (IHC)
^a^ Expression should be at least similar to that seen in stage I B-cell precursors or mature B cells. ^b^ At least a fraction of the blasts should express CD3 intensity similar to that of background mature T cells.	

## Abbreviations

WHO-HEM5	The 5th edition of the World Health Organization Classification of Tumors of the Hematopoietic and Lymphoid Tissues
ICC	The International Consensus Classification
ALAL	Acute leukemia of ambiguous lineage
MPAL	Mixed-phenotype acute leukemia
AUL	Acute undifferentiated leukemia
AML	Acute myeloid leukemia
ALL	Lymphoblastic leukemia
AML-MR	A subtype of AML with myelodysplasia-related (MR) changes
MPO	Myeloperoxidase
Lineage-defining markers	Markers used to assign the lineage differentiation for diagnosis of MPAL by the WHO-HEM5/ICC, such as CD3 and MPO
Lineage-associated markers	Markers commonly expressed but not specific for the lineage commitment, such as CD13 and CD33
